# Structural Health Monitoring in Composite Structures by Fiber-Optic Sensors †

**DOI:** 10.3390/s18041094

**Published:** 2018-04-04

**Authors:** Alfredo Güemes, Antonio Fernández-López, Patricia F. Díaz-Maroto, Angel Lozano, Julian Sierra-Perez

**Affiliations:** 1 Department Aeronautics, Polytechnic University of Madrid, 28040 Madrid, Spain; antonio.fernandez.lopez@upm.es (A.F.-L.); p.fernandezdm@gmail.com (P.F.D.-M.); angel.lmrtn@gmail.com (A.L.); 2 Ingeniería Aeroespacial, Universidad Pontificia Bolivariana, Medellín 050031, Colombia; julian.sierra@upb.edu.co

**Keywords:** structural health monitoring (SHM), distributed sensing, principal component analysis (PCA)

## Abstract

Fiber-optic sensors cannot measure damage; to get information about damage from strain measurements, additional strategies are needed, and several alternatives are available in the existing literature. This paper discusses two independent procedures. The first is based on detecting new strains appearing around a damage spot. The structure does not need to be under loads, the technique is very robust, and damage detectability is high, but it requires sensors to be located very close to the damage, so it is a local technique. The second approach offers wider coverage of the structure; it is based on identifying the changes caused by damage on the strain field in the whole structure for similar external loads. Damage location does not need to be known a priori, and detectability is dependent upon the sensor’s network density, the damage size, and the external loads. Examples of application to real structures are given.

## 1. Introduction

It is important to recognize that measuring strains is not the same as detecting damage. Damage is not a physical parameter, it is just a local change in the material’s properties or at the structure boundaries (a crack is simply a new boundary) that degrades structural performance [1]. A crack may be the failure initiation point and may drop the strength of the structure by a large percentage, but before catastrophic failure, it produces negligible changes in most of the structure’s parameters (natural frequencies, global strain fields, and so on). Damage can only be detected by comparing the responses of the structure, acquired by sensors, before and after it occurs. Consequently, we cannot expect to have “damage sensors”, we can only get information about damage by processing and comparing the raw signals received from the sensors before and after damage, in an attempt to identify the “features”, or parameters, that are sensitive to minor damage and that can be distinguished from the response to natural and environmental disturbances. In this sense, structural health monitoring (SHM) is always sensors plus damage detection algorithms, and in some cases, such as comparative vacuum monitoring (CVM), the algorithm may be quite simple (loss of vacuum in some channels).

The first attempts at damage detection with optical fibers dates back to 1990, under the heading “structures with nerves of glass”, by checking the continuity of the optical fiber [2]. The approach was not robust enough, and this research line was discontinued. Fiber Bragg grating (FBG) started to be used as strain sensors embedded in composite structures around 1995, and some articles appeared around 2000 looking for changes in the spectrum of the reflected peak as an indicator of damage, when the damage was happening on just the position of the embedded FBG. Again, the procedure cannot be extended to a general case of damage detection in structures. 

**Figure 1:**
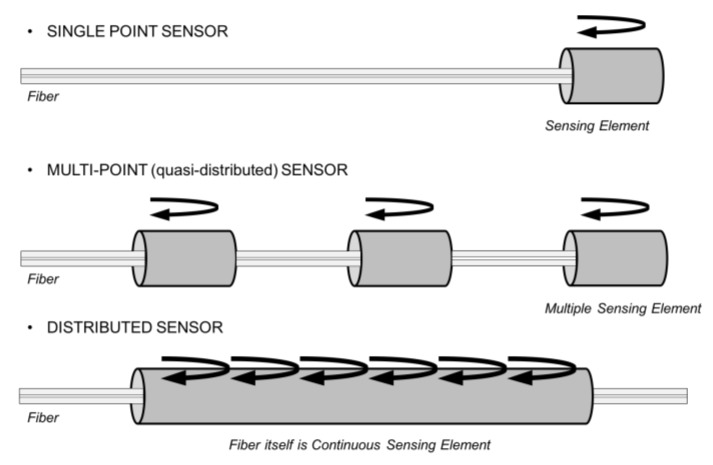
Topology of fiber-optic sensors.

Fiber-optic sensors (FOS) offer a very small size: the optical fiber has a diameter of 150 microns when coated with polyimide, so it can be embedded within the composite material during manufacturing. Other benefits of FOS are EMI/RFI immunity, wide temperature range, very long cabling if needed because of the low attenuation, and multiplexing capability (several sensors on the same optical fiber). As sketched in Figure 1, three topologies are possible:

- Point sensor: This detects measurand variation only in the vicinity of a single sensor; e.g., micromirror at fiber tip. This is mostly used for chemical sensors, but also for extrinsic Fabry-Perot interferometer.
- Multipoint sensor: Multiple localized sensors are placed at intervals along the fiber length, e.g., FBG (typical sensor length 10 mm), about 10 sensors/fiber if multiplexed by wavelength, up to 1000 sensors by using optical frequency domain reflectometry (OFDR).
- Distributed sensor: Sensing is distributed along the length of the fiber; the optical fiber works simultaneously to transmit the information and sense the local external variables (temperature, strain).

Fiber-optic sensors (FOS) have reached maturity as strain/temperature sensors. It can be said that their technology readiness level is about 8–9, they have already been demonstrated in real aircraft, and they are routinely used in many other industrial applications, like monitoring oil wells. An excellent 50-page review on FOS technologies has recently been done [3], including more than 170 references, so a long introduction describing FOS is not needed, and readers are referred to that review or other similar documents [4,5,6]. 

Despite the very large number of publications dealing with it, damage detection with FOS is less mature than strain sensing.

Currently the most widely known approach is based on embedding FBG sensors to detect ultrasonic waves travelling through laminates, either in passive mode (acoustic emission) or in active mode, combined with piezoelectric wafers as Lamb wave emitters. A quite clear explanation of the technique can be found in [7]. Several authors have also worked on this concept, developing advanced interrogation systems, some now commercially available. This hybrid PZT/FBG technique has the same limitations as the all-PZT technique: it deals with elastic waves propagating through the structure. It works very well on flat laminates or very simple structures such as pipes, but real structures have stiffeners and local reinforcements, which produce multiple reflections of the travelling waves, limiting the range of inspection and adding complexity to the received signals. 

A review of strain-based damage detection strategies, mainly oriented to civil structure applications, was done in [8], but was limited to vibration-based methods, which inherently have the advantage of a global survey of the whole structure, and the limitation that damage needs to be large enough to modify the modal shapes. The authors concluded that, at least for beam-like and truss structures, strain modal shapes are more sensitive to damage than modal displacements [9]; damage indexes were proposed, but few experimental results were presented. A similar approach was applied to composite stiffened panels, including numerical simulations and experimental tests [10,11], but the accuracy of those strategies for damage quantification has yet to be verified. 

The first procedure to be discussed in this paper deals with detection of new internal strains that appear in a composite structure as a consequence of damage; these new strains are concentrated around the damaged area, and will be detected if some strain sensors are located there. A few centimeters away from the damaged area there will not be any strain changes, and consequently nothing would be detected. The approach is quite robust; delamination always produces strong local change in strains, but the area under supervision is limited to the area covered by the strain sensors, so the technique is quite local, similar to CVM. It can be done with FBGs, but even with a very dense array of sensors, only a small area can be supervised. A technique available since 2005 for high-spatial-resolution distributed sensing (known as optical frequency domain reflectometry, OFDR, or optical backscatter reflectometry, OBR) can get strain readings all along the optical fiber, allowing for wider coverage. Results of its application to the surveillance of composite door surroundings is given. We call this approach detection of damage-induced strains.

The former procedure may be applied to unloaded structures, so the strain readouts are zero everywhere (baseline) except at the damage area. For the second procedure, when the structure is submitted to an external load, each strain sensor at the structure will give a readout for the local strain at that sensor position, with a linear dependence on the external load. Local damage will produce a change in local stiffness, and consequently a change at the load paths, and on the readouts at each strain sensor (for the same external loads); nevertheless, the changes will be so small that they can hardly be detected, and very precise algorithms are needed to distinguish them. This is the basic principle for the second approach to be discussed in the paper, sometimes referred as strain mapping. 

Some of the results presented here were formerly published; the paper is a review of previous works, the organic reconsideration of which gives a comprehensive presentation and comparison of the two different methods.

## 2. Detection of Damage-Induced Strains 

To be effective, this technique requires distributed sensing, which means getting the strains all along the optical fiber. Several kinds of fiber-optic distributed sensing systems are available, depending on the wavelength they are working with [12]. Table 1 summarizes their performance. The Rayleigh system working with OFDR is the only one to offer spatial resolution in the millimeter range, as is needed for aeronautic applications; for civil engineering applications, a long measurement range may be preferred, which may drive other choices. Performance evolves quickly, so this table must be taken with caution.

Distributed sensing has opened new possibilities for the instrumentation of structural tests, particularly for very large structures like civil engineering structures [13,14,15] and wind turbine blades [16]. Again, getting strains is not the same as getting damage information, even though in concrete structures, cracks are easily identified as the points with very high strain readouts. 

### 2.1. Detection of Delaminations Caused by Impact

Impact damage is considered to be the highest threat to composite structures during their service life. Low/medium energy impact (called barely visible impact damage, BVID) does not leave any external visible marks, but causes internal delaminations that drop the compressive strength by nearly 50%. Such impact needs to be identified and repaired as soon as possible to avoid growth of the damaged area under repeated loads. 

**Figure 2:**
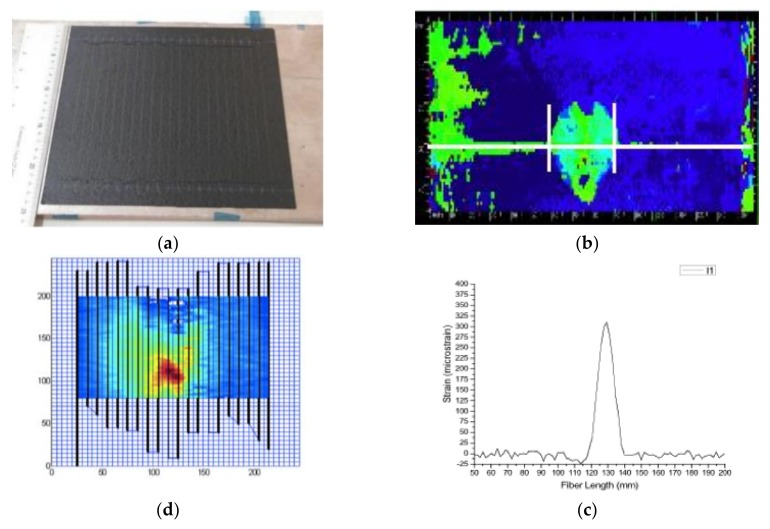
Detection of delamination caused by impact. (**a**) Composite laminate with optical fiber following parallel paths; (**b**) ultrasonic C-scan of the impacted laminate; (**c**) strains measured by optical backscatter reflectometry (OBR) along the white line of the upper figure; (**d**) strain plotting at the delaminated area, obtained by parallel optical fibers.

A 16-ply crossply CFRP laminate was built from UD prepreg material by out of autoclave (OOA) procedures. A polyimide-coated optical fiber was embedded inside the laminate during layup (Figure 2a). CFRP means Carbon Fiber Reinforced Plastic. UD stands for Unidirectional.

The laminate was impacted by a drop weight test and a delamination was produced, as verified by ultrasonic C-scan (Figure 2b, green spot). The white line shows the position of the optical fiber, and the lower image (Figure 2c) shows the strains measured by the optical fiber along this line. The appearance of residual strains at the delaminated area can be seen. It is worth mentioning that strains caused by damage are significant, with a peak of 300 microstrains, and the delaminated length is perfectly depicted, 25 mm. In fact, the delaminated area can nearly be plotted if the optical fiber follows a crooked path, with parallel fibers every 5 mm. The strain field map of the area can be obtained with relatively high accuracy (Figure 2d).

### 2.2. Detection of Delaminations at Laminate Edges

The former approach can be used for SHM of similar structures, like small cylindrical pressure vessels [17], or for structural details, like monitoring stringer debondings [18]; it has also been used to monitor bonded and bolted joints ([19,20], respectively). But for practical reasons, the whole surface of the aircraft cannot be covered with a continuous optical fiber; the maximum inspectable length is about 100 m. This concept is useful by reducing the covered area to critical regions with a higher risk of damage.

Laminate edges, such as surroundings of cargo doors and manholes, are areas at high risk for accidental impact, and consequently require more frequent inspection; a permanent automated inspection system is highly desirable. The following experiments were done to demonstrate the validity and reliability of the approach; full details are given in [21]. 

**Figure 3:**
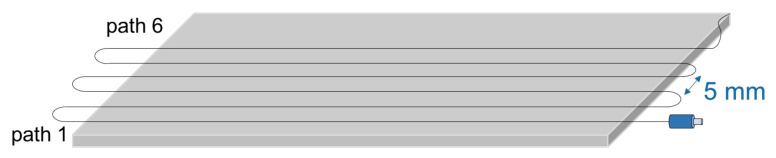
Laminate with optical fiber bonded at the surface for edge delamination tests.

Several identical CFRP 16-ply laminates were built from UD prepreg material by OOA procedures, with the layup (0_4_, 90_4_)s. This special layup sequence was used for simplicity, to have only two delamination interfaces; nevertheless, the concept also works for any general laminate. Dimensions of the cured laminate were 200 mm × 100 mm. An optical fiber was bonded at the surface of the cured laminate, as sketched in Figure 3.

**Figure 4:**
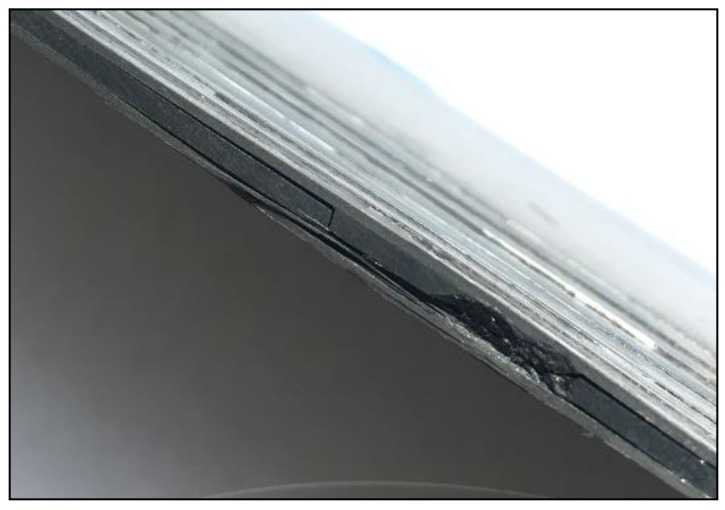
Delamination of (0_4_, 90_4_)s CFRP laminate after a 5 joule on-edge impact.

**Figure 5:**
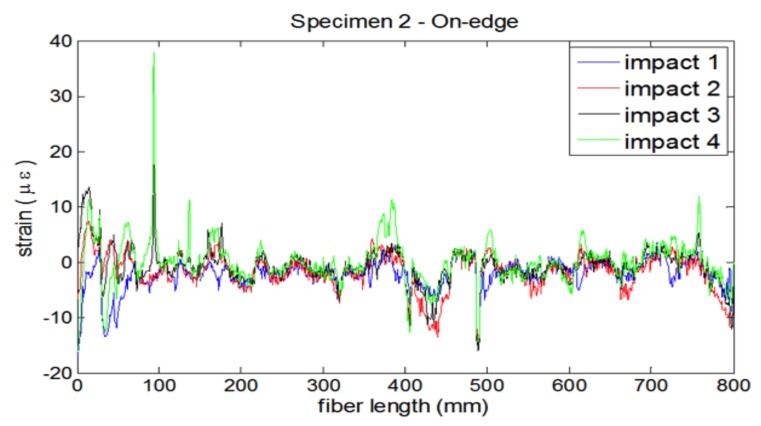
Strains recorded by OBR on the optical fiber after successive impacts of increasing energy, from 2 to 5 joules. The two identifiable peaks recorded at 100 and 400 mm are for the first and second loops of the optical fiber, at a distance to the edge of 5 and 10 mm, respectively.

The laminates were submitted to impacts of controlled energy by using a drop weight test machine both perpendicular to the laminate and in the on-edge direction. The energy was gradually increased until visible damage was produced (Figure 4), and the residual strains were recorded after every impact (Figure 5).

Similar findings were obtained when impacts were done at the direction normal to the laminate. For these tests, an embedded optical fiber was used, located at the second ply of the surface opposed to the impact. The energy needed to cause a BVID was slightly higher than in the former case. These results show that the technique is highly reliable, as far damage happening on the optical fiber path, and that a system is available to get the strains all along the fiber with adequate spatial resolution. 

## 3. Detection of Damage by Strain Mapping 

There have been different attempts to use strain data collected after static or dynamic loading of the structure to derive damage information. It is suspected, and finite element models confirm, that local damage changes the strain readouts slightly, more significantly at the sensors located closer to the damage region. As will be shown below, for realistic structures, a large number of sensors are needed, each sensor producing data for each load case or load increment; so even for simple experiments, huge datasets in the Gigabit range will be generated, which contain redundant and repeated information accompanied by noise. 

**Figure 6:**
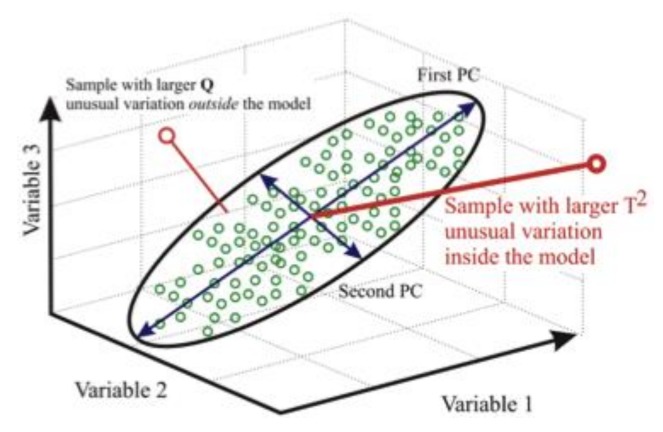
Schematics of the principal component analysis (PCA) algorithm. First, identify a new coordinate system that reduces the dimensionality of the dataset. Second, identify whether or not new data fits inside the former reference system.

Many algorithms are available to handle large datasets [22]; one of the simplest and most effective approaches is called principal component analysis (PCA). PCA is a simple and nonparametric method of extracting relevant information from confusing datasets. It provides hints on how to reduce a complex dataset to a lower dimension, revealing some hidden structure/patterns or abnormal data. This is done by converting a set of data of possibly correlated variables into a set of values of linearly uncorrelated variables called principal components (Figure 6). Textbooks and software tools are available explaining the use of PCA [23], and related articles dealing with the application of PCA to SHM [24], so only a brief explanation is given here. The steps to follow are:

1. Organize the data set as an n × m matrix, where n is the number of tests (each load case or load increment is a new test) and m is the number of measured variables (sensors): X.
2. Normalize the data to have zero mean and unity variance.
3. Calculate the eigenvectors-eigenvalues of the covariance matrix: C = X X^T^.
4. Keep only the first eigenvectors as the principal components: Baseline.
5. Project any new collected data into the former Baseline.
6. Identify whether new data follow global trends (damage index).

PCA belongs to the group of “data-driven” SHM methods, different from “model-based” methods like vibration analysis, so an understanding of either the physical meaning of the new variables or a detailed modeling of the structure is needed. Also, the five levels for structural health monitoring must be pointed out:

1. Identification of damage occurrence
2. Localization
3. Identification of damage type
4. Quantification of damage
5. Prediction of residual strength

This technique may only provide an alert for damage occurrence; it does not seem to be a main limitation, since once damage is known to happen, it can be located by checking which area has the largest strain changes. 

Two examples are given for the application of this method to realistic structures.

### 3.1. Damage Detection for a CFRP Isogrid Structure

The concept of isogrid or lattice structures has been widely explored for space applications, both with and without attached skins. This kind of structure has an inherent high mechanical efficiency, particularly in withstanding compressive loads. 

**Figure 7:**
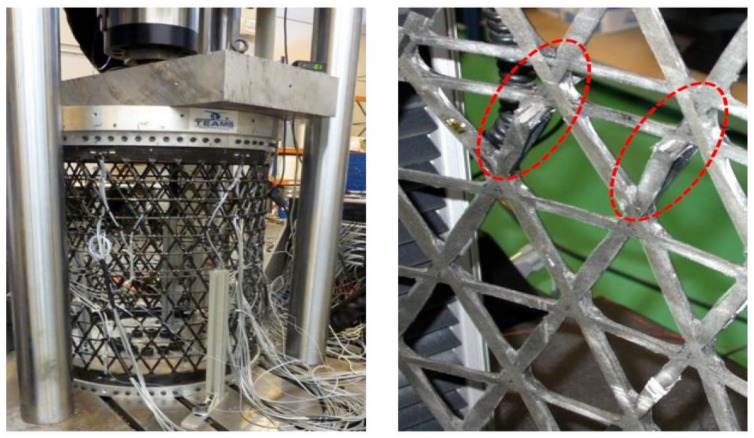
(**Left**) Isogrid structure under compression test. (**Right**) Detail of broken bars after exceeding the maximum load.

**Figure 8:**
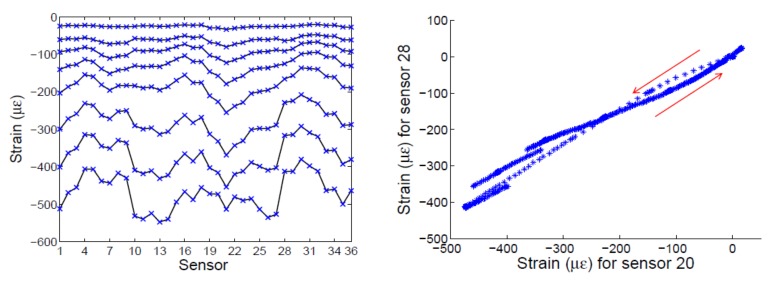
(**Left**) Isogrid structure under compression test. (**Right**) Strain data under increasing loads, showing nonuniformity due to manufacturing imperfections and nonlinear behavior.

A large structure, 1100 mm in height and 800 mm in diameter, has been manufactured by an automatic tape-laying process using high modulus graphite fiber and an autoclave curable resin system. It was instrumented with 36 FBGs and tested to fail under compressive loads (Figure 7). The first failure happened at –330 kN, with some broken bars, but the structure retained its load-carrying capability. Figure 8 shows the strains acquired during the test, which gives evidence that slight manufacturing imperfections caused an uneven strain distribution and a nonlinear response under load/unload conditions.

**Figure 9:**
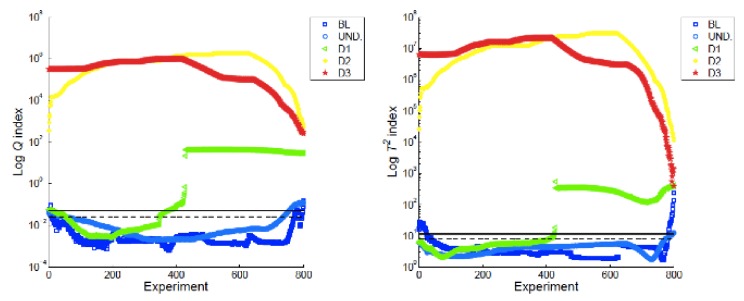
PCA algorithm applied to isogrid strain data. Blue lines are damage indexes calculated from strains acquired on the undamaged structure. Each experiment means new data acquisition (load level); green lines correspond to data taken under increasing loads. First failure happened at test 400.

The application of the PCA algorithm to the former dataset is straightforward, with quite good results (Figure 9). It is worth mentioning that there is no need to prepare the data or a finite element model, only to arrange the X matrix (36 × 800). There were 36 sensors, and 800 measurements were taken to generate the baseline. The next measurements, projected on this baseline, easily discriminated the load that produced the first failure, and after that, the next measurements had a clearly detectable damage index.

### 3.2. Damage Detection for a Wind Turbine Blade

**Figure 10:**
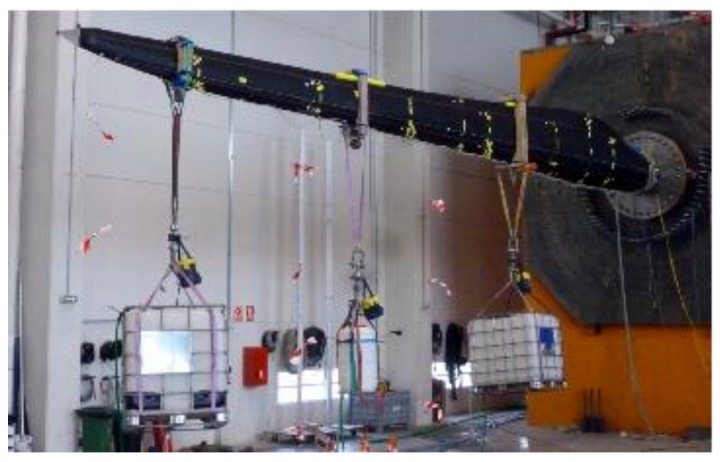
PCA algorithm applied to isogrid strain data.

Compared to the former case, this experiment has two added difficulties: First, the load cases are not as simple as for the isogrid structure, always uniformly distributed compressive loads of increasing value. As shown in Figure 10, different load cases, or distributions of weights where loads were applied, can be done. As a consequence, it will be found that there is not a single dominant principal component; the first three components play similar roles. 

**Figure 11:**
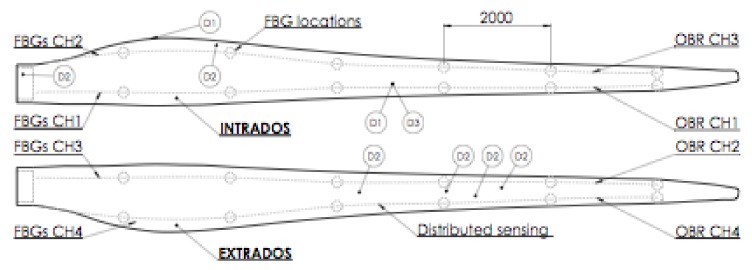
Blade sketch indicating sensors and damage positions.

The second difficulty comes from the nature of the inflicted damage. To do a representative experiment, the typical damage that happens to a wind turbine blade (WTB) was reproduced; that is, a partial debonding of the shells at the trailing edge, as marked in Figure 11. A blade 13.5 m long, manufactured in the conventional way as a long spar with two bonded shells, was instrumented with four optical fibers with six FBGs each, regularly spaced. For a cantilever beam under flexural loads, partial trailing-edge debonding changes the torsional stiffness, but does not strongly alter the bending stiffness.

As before, the structure was submitted to loads of increasing levels, and strains were recorded, first without any damage to obtain the baseline, and then after artificial damage. The damage index was calculated, and it was found that when debonding was 100 mm long, it was clearly detectable.

**Figure 12:**
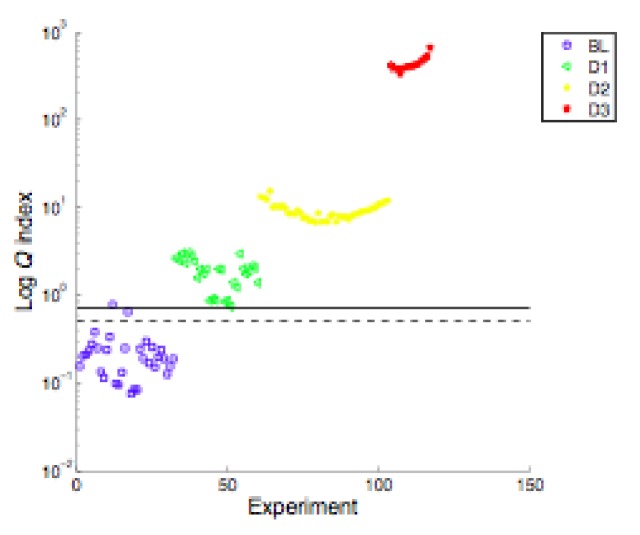
PCA algorithm applied to wind turbine blade.

Results are shown in Figure 12, green dots. Obviously, when new damage was inflicted on the main spar, as a load-carrying member, it was more easily detectable. 

Further details on this and the prior experiment can be found in Dr. Sierra’s thesis [25], including improvements to the PCA algorithm, such as the nonlinear PCA, the use of neural networks to classify load cases, and the results obtained for the same experiment when a strain distributed sensing system was used instead of multipoint FBGs (many more strain measurements were collected, thus higher resolution was achieved).

## 4. Discussion

Two independent methods have been described and experimentally validated. The first one is based on detecting new strains appearing as a consequence of damage and requires a sensor to be located just there; it is a very robust technique and very simple to apply, but the area of damage detection is limited to the fiber path. It should be considered, as it has similar damage-detection qualities as CVM, which currently is the only certified SHM technology for aircraft structures. The examples given demonstrate the high resolution of the technique, able to detect delaminations as small as 5 mm (twice the resolution of the OFDR distributed interrogation systems).

The second approach offers full coverage of the whole structure. Two examples are given to demonstrate that the algorithm PCA is easy to apply, and that the damage index Q consistently identifies the damaged structure. Nevertheless, it is recognized that the minimum detectable damage size is still too large, so it needs to be improved. As stated, we used one of the most basic tools for multivariate analysis. Currently, we are working on more elaborate tools to improve the resolution, and for other realistic applications.

## Figures and Tables

**Table 1:** Comparison of distributed fiber-optic sensor systems.

	Rayleigh	Raman	Brillouin	Distributed Acoustic Sensing
Domain	OFDR	OTDR	BOTDR, BOTDA	φ-OTDR
Sensing Parameter	Strain Temperature	Temperature	Strain Temperature	Vibrations, Acoustic Signals
Maximum Distance	70–100 m	20 km	10 km	40 km
Spatial Resolution	5 mm	1–2 m	10 cm	1–2 m
Strain Accuracy	1 με	1 με	25 με	None
Suppliers	LUNA, 4DSP	Halliburton Co. Sensornet Ltd. AP Sensing	OZ Optics, Omnisens SA, Neubrex	OptaSense, Xilixa
